# West Nile Virus Isolation in Human and Mosquitoes, Mexico

**DOI:** 10.3201/eid1109.050121

**Published:** 2005-09

**Authors:** Darwin Elizondo-Quiroga, C. Todd Davis, Ildefonso Fernandez-Salas, Roman Escobar-Lopez, Dolores Velasco Olmos, Lourdes Cecilia Soto Gastalum, Magaly Aviles Acosta, Armando Elizondo-Quiroga, Jose I. Gonzalez-Rojas, Juan F. Contreras Cordero, Hilda Guzman, Amelia Travassos da Rosa, Bradley J. Blitvich, Alan D.T. Barrett, Barry J. Beaty, Robert B. Tesh

**Affiliations:** * Universidad Autonomo de Nuevo Leon, San Nicolas de Los Garza, Nuevo Leon, Mexico;; † University of Texas Medical Branch, Galveston, Texas, USA;; ‡ Servicios de Salud de Sonora, Hermosillo, Sonora, Mexico;; § Colorado State University, Fort Collins, Colorado, USA

**Keywords:** West Nile virus, arbovirus, vector-borne disease, infectious disease, viral disease, epidemiology, viral ecology, Flavivirus, dispatch

## Abstract

West Nile virus has been isolated for the first time in Mexico, from a sick person and from mosquitoes (*Culex quinquefasciatus*). Partial sequencing and analysis of the 2 isolates indicate that they are genetically similar to other recent isolates from northern Mexico and the western United States.

Several recent reports (*1**–**7*) have documented the widespread geographic distribution of West Nile virus (WNV) in Mexico, but until now, no autochthonous human cases of illness due to this virus have been reported from the republic. Likewise, limited entomologic surveillance has been conducted in Mexico, and no information is available on the actual mosquito vectors of WNV in the republic. All Mexican WNV isolates studied to date have come from dead equines or birds (*2**,**7**,**8*). We report the first isolations of WNV from a sick person and from a pool of *Culex quinquefasciatus* mosquitoes and describe their phylogenetic relationship to other representative WNV strains from the United States and Mexico.

## The Study

Mosquitoes were collected from June to September 2003 at the Ejido Francisco Villa, Municipality of Pesqueria, State of Nuevo Leon (25°47´N, 100°03´W), with CDC-type light traps baited with dry ice and mechanical aspiration from resting sites on vegetation and in houses. The area is located ≈40 km northeast of Monterrey and consists of mixed suburban housing and agriculture. Average annual rainfall in the region is 550 mm; the mean annual temperature is 28°C. After collection, the mosquitoes were placed on dry ice for transport back to the Medical Entomology Laboratory, Faculty of Biological Sciences, Autonomous University of Nuevo Leon, Monterrey, where they were separated into pools of ≈10 insects each, based on species, date, and method of collection (Table 1). The mosquitoes were stored in a mechanical freezer at –70°C and later transported on dry ice to the University of Texas Medical Branch (UTMB) to be processed for virus isolation. A total of 2,297 mosquitoes, representing 4 genera and 11 species, were tested in 238 pools (Table 1). Individual mosquito pools were titrated manually in sterile, Ten Broeck tissue grinders containing 1.0 mL of phosphate-buffered saline, pH 7.4, containing 30% fetal bovine serum and antimicrobial agents (penicillin, streptomycin, and amphotericin). The resultant suspension was centrifuged at 12,000 rpm for 5 min; then 200 μL of the supernatant was injected into a flask culture of Vero cells. After the solution was absorbed for 1 h at 37°C, maintenance medium (*9*) was added; cultures were maintained in an incubator at 37°C and examined daily for evidence of viral cytopathic effect (CPE) for 14 days.

**Table 1:** Summary of mosquitoes collected in Nuevo Leon, Mexico, during the summer of 2003 and tested for West Nile virus

Genus and species	No. pools	No. mosquitoes
*Aedes aegypi*	39	399
*Ae. vexans*	1	10
*Ochlerotatus taeniorynchus*	15	146
*Anopheles pseudopunctipenis*	1	8
*An. quadrimaculatus*	1	2
*Culex coronator*	13	118
*Cx. quinquefasciatus*	81	798
*Psorophora ciliata*	5	22
*Ps. confinnis*	2	8
*Ps. cyanescens*	9	89
*Ps. ferox*	70	697

A single pool of *Cx. quinquefasciatus* yielded a virus isolate, designated NL-54, which produced CPE on approximately day 7. The isolate was identified as WNV by immunofluorescence, hemagglutination-inhibition (HI) test, complement-fixation test, VecTest WNV/SLE antigen assay (Medical Analysis Systems, Camarillo, CA, USA), and reverse transcription–polymerase chain reaction (RT-PCR) (*9**,**10*).

The WNV human isolate was from a 62-year-old Mexican woman living in the municipality of Etchojoa (near Ciudad Obregon) in Sonora State. The patient had no history of travel during the preceding 2 months. She visited a local hospital in July 2004 with symptoms of fever, headache, vomiting, arthralgias, and myalgia. Her temperature was 38°C upon examination, and no neurologic symptoms were noted. An acute-phase blood sample was obtained, and a presumptive diagnosis of dengue fever was made. The patient was sent home and subsequently completely recovered. When RT-PCR using dengue primers was negative on the acute-phase serum, a culture was performed. WNV was isolated from the sample at the State Public Health Laboratory in Sonora and at UTMB, upon culture in Vero cells. HI tests conducted on the acute-phase serum at UTMB with West Nile, St. Louis encephalitis, yellow fever, dengue 1, and dengue 2 viral antigens were negative, which indicated that the patient had no preexisting flavivirus antibodies. An immunoglobulin (Ig) M enzyme-linked immunosorbent assay (*11*), performed on the acute-phase specimen and a 30-day convalescent-phase serum specimen in Sonora, demonstrated seroconversion and the presence of WNV-reactive IgM antibodies in the convalescent-phase serum sample.

Viral RNA was extracted from the 2 WNV strains after a single Vero cell passage directly from 140 μL of the infected cell culture supernatants, using the QIAamp viral RNA extraction kit (*12*). RT-PCR was performed by using 3 primer pairs to amplify the entire prM-E genes of each WNV isolate as previously described (*12*). PCR products were gel purified with the QIAquick kit (Qiagen, Valencia, CA, USA) according to the manufacturer's protocol, and the resulting template was directly sequenced with the amplifying primers. Sequencing reactions were performed as described previously (*8*). Analysis and assembly of sequencing data were performed with the Vector NTI Suite software package (Informax, Frederick, MD, USA). Nucleotide and deduced amino acid sequences of the 2004-nucleotide region representing the prM-E genes from each isolate were aligned with the AlignX program in the Vector NTI Suite and compared to sequences of selected North American WNV isolates for which the prM-E genes were available in GenBank. Phylogenetic trees were constructed by Bayesian analysis with the program MRBAYES, version 2.0 (*13*), with the Metropolis-coupled, Markov chain, Monte Carlo algorithm run with 4 chains over 150,000 generations under a general time-reversible model with a burn-in time of 50,000 generations. Rate heterogeneity was estimated by using a γ distribution for the variable sites. The Bayesian consensus tree was compared to trees generated by neighbor-joining, maximum parsimony, and maximum likelihood analyses using PAUP, version 4.0b10 (*14*), and each method generated trees with the same overall topology. The consensus phylogram of the 40 WNV isolates generated by Bayesian analysis (*13*) is shown in the Figure, with confidence values at relevant nodes to demonstrate statistical support for each clade.

**Figure:**
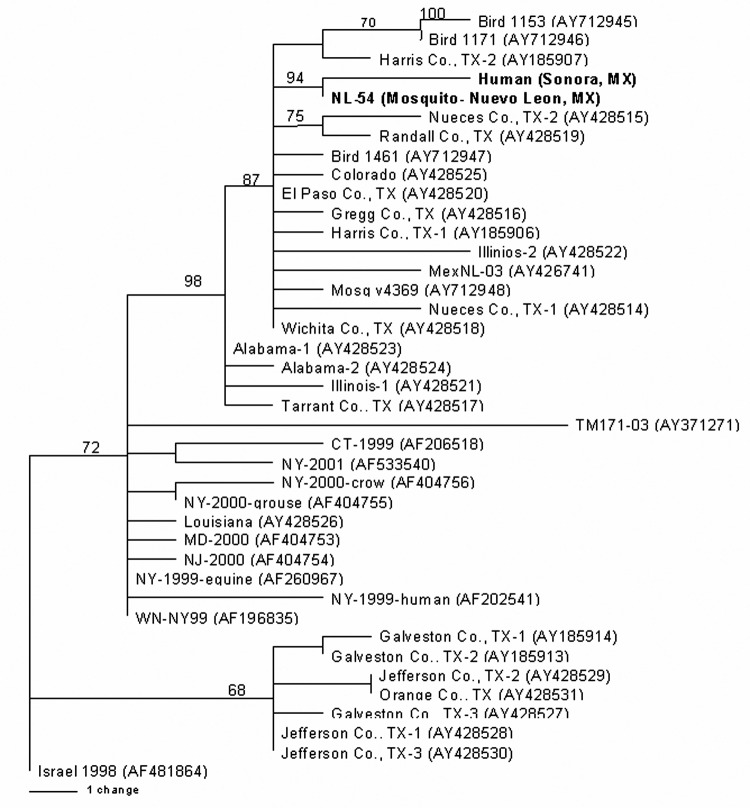
Figure. Phylogram of 2 West Nile viruses (WNV) isolated from a mosquito pool and human serum in Mexico (shown in **bold**). The phylogenetic tree was generated by Bayesian analysis of a 2004-nucleotide region of the prM and E genes of 40 WNV isolates rooted by the most closely related Old World strain, Israel 1998. Bayesian confidence values are shown to provide statistical support for each clade.

## Conclusions

Comparison of the 2 Mexican isolates, NL-54 (GenBank accession no. AY963775) and human Sonora (GenBank accession no. AY963774), to the prototypical North American WNV isolate, WN-NY99 (GenBank accession no. AF196835), 2 previous Mexican isolates, MexNL-03 (GenBank accession no. AY426741) (*7*) and TM171-03 (GenBank accession no. AY371271) (*2*), and an isolate collected in Harris County, Texas, in 2002 (GenBank accession no. AY185906) (*15*) indicated nucleotide and deduced amino acid differences and similarities among each of the isolates. Table 2 shows the positions at which nucleotide and amino acid substitutions were found. Both the Mexican mosquito and human isolates reported herein shared a nucleotide mutation at position 660 (C to U) of the prM gene and 2 mutations at positions 1442 (U to C) and 2466 (C to U) of the E gene. Each of these 3 mutations was shared with a 2003 horse strain from Nuevo Leon (MexNL-03) (*7*) and a 2002 bird isolate from Harris County, Texas (TX-1) (*15*). The mutation at nucleotide 1442 also represented a deduced amino acid substitution in the envelope protein (V159A). The Mexican mosquito and human isolates reported herein shared a unique mutation at genomic position 1320 (A to G) in the E gene. The human isolate also had 3 additional mutations in the E gene at positions 1074 (G to A), 1656 (U to C), and 1974 (C to U). Each of the additional nucleotide mutations was silent. The nucleotide mutations at nucleotide positions 660, 1442, and 2466 have also been described in most WNV isolates sequenced from Texas, Illinois, and Colorado in 2002 (*12*). This finding suggests that isolates obtained from northern states of Mexico (i.e., Nuevo Leon and Sonora) were derived from WNV strains circulating in the western United States. Only a single mutation at position nucleotide 2466 was shared by these 2 isolates and a 2003 bird isolate from Tabasco State (TM171-03). This finding supports results from earlier studies that suggest separate introductions of WNV into Mexico (*2**,**7*). Phylogenetic trees generated by a number of methods indicate that the recent Mexican mosquito and human isolates belong to the clade comprised of WNV isolates collected outside the northeastern United States after 2001, with the exception of isolates collected along the southeast coast of Texas. (Those isolates constitute a separate, sister clade relative to all other North American WNV isolates sequenced to date [Figure].). Because of a shared mutation between the recent Mexican mosquito and human isolates, these 2 virus strains constitute a distinct subclade within the larger US 2002 clade that is supported by strong Bayesian confidence values (94%). The accumulation of 3 additional nucleotide mutations in the 2004 Mexican human isolate is illustrated by longer branch lengths in comparison to the 2003 mosquito pool isolate NL-54, which suggests the continued microevolution of WNV in Mexico from year to year.

**Table 2:** Nucleotide and deduced amino acid differences in the prM-E genes of Mexican and Texas isolates compared to West Nile virus strain WN-NY99 (382-99)

Strain	Nucleotide (amino acid) substitutions in prM and E genes (nt 466–2469)*																		
	483	549	660	858	887	1074	1137	1179	1320	1356	1432	1442	1626	1656	1974	2328	2388	2392	2466
WN-NY99 (AF196835)†	C	U	C	C	U (Ile)	G	C	A	A	C	U (Ser)	U (Val)	C	U	C	C	C	G (Ala)	C
TM171-03 (AY371271)	U	‡		U	C (Thr)		U				C (Pro)		U			U	U		U
MexNL-03 (AY426741)		C	U					G		U		C (Ala)							U
NL-54 (Mosquito, MX)			U						G			C (Ala)							U
Human (Sonora, MX)			U			A			G			C (Ala)		C	U				U
Harris Co., TX-1 (AY185906)			U									C (Ala)						A (Thr)	U

*Nucleotide numbers correspond to WN-NY99; amino acid substitutions are in brackets. †GenBank accession number. ‡Blank entries indicate no nucleotide or amino acid substitutions.

Our patient represents the first reported autochthonous human case of confirmed WNV infection in Mexico. The paucity of human cases reported to date from Mexico is curious for several reasons: 1) a large number of cases are reported from the United States, 2) available evidence indicates that WNV is now widely distributed in Mexico (*1**–**7*), 3) most of the WNV virus strains circulating in the republic are genetically similar to those in the United States (Figure). One explanation for this difference could be the failure of local health personnel to recognize the various clinical forms of WNV infection. As illustrated by our patient, West Nile fever can easily be mistaken for dengue fever. A second reason may be the difficulty of making a serologic diagnosis of WNV infection among persons living in geographic regions where several different flaviviruses circulate, and people have multiple flavivirus infections (*11*). A third and related possibility is that WNV infection may be less severe in persons with preexisting heterologous flavivirus antibodies (*11*).

## References

[1] Blitvich BJ, Fernandez-Salas I, Contreras-Cordero JF, Marlenee NL, Gonzalez-Rojas JI, Komar N, Serologic evidence of West Nile virus infection in horses, Coahuila State, Mexico. Emerg Infect Dis. 2003;9:853–6. 12890327 10.3201/eid0907.030166 PMC3023419

[2] Estrada-Franco JG, Navarro-Lopez R, Beasley DWC, Coffey L, Carrara A-S, Travassos da Rosa A, West Nile virus in Mexico: serologic evidence of widespread circulation since July 2002. Emerg Infect Dis. 2003;9:1604–7. 14720402 10.3201/eid0912.030564 PMC3034333

[3] Lorono-Pino MA, Blitvich BJ, Farlan-Ale JA, Puerto FI, Blanco JM, Marlenee NL, Serologic evidence for West Nile virus infection in horses, Yucatan State, Mexico. Emerg Infect Dis. 2003;9:857–9. 12890328 10.3201/eid0907.030167 PMC3023444

[4] Ulloa A, Langevin SA, Mendez-Sanchez JD, Arredondo-Jimenez JI, Raetz JL, Powers AM, Serologic survey of domestic animals for zoonotic arbovirus infections in the Lacandon Forest region of Chiapas, Mexico. Vector Borne Zoonotic Dis. 2003;3:3–9. 10.1089/153036603765627406 12804375

[5] Fernandez-Salas I, Contreras-Cordero JF, Blitvich BJ, Gonzalez-Rojas JI, Cavazos-Alvarez A, Marlenee NL, Serologic evidence of West Nile virus infection in birds, Tamaulipas State, Mexico. Vector Borne Zoonotic Dis. 2003;3:209–13. 10.1089/153036603322662192 14733673

[6] Farfan-Ale JA, Blitvich BJ, Lorono-Pino MA, Marlenee NL, Rosado-Paredes EP, Garcia-Rejon JE, Longitudinal studies of West Nile virus infection in avians, Yucatan State, Mexico. Vector Borne Zoonotic Dis. 2004;4:3–14. 10.1089/153036604773082942 15018768

[7] Blitvich BJ, Fernandez-Salas I, Contreras-Cordero JF, Lorono-Pino MA, Marlenee NL, Diaz FJ, Phylogenetic analysis of West Nile virus, Nuevo Leon State, Mexico. Emerg Infect Dis. 2004;10:1314–7. 15324558 10.3201/eid1007.030959 PMC3323327

[8] Beasley DWC, Davis CT, Estrada-Franco J, Navarro-Lopez R, Campomanes-Cortes A, Tesh RB, Genome sequence and attenuating mutations in West Nile virus isolate from Mexico. Emerg Infect Dis. 2004;10:2221–4. 15663867 10.3201/eid1012.040647 PMC3323401

[9] Lillibridge KM, Parsons R, Randle Y, Travassos da Rosa APA, Guzman H, Siirin M, The 2002 introduction of West Nile virus into Harris County, Texas, an area historically endemic for St. Louis encephalitis. Am J Trop Med Hyg. 2004;70:676–81. 15211013

[10] Tesh RB, Parsons R, Siirin M, Randle Y, Sargent C, Guzman H, Year-round West Nile virus activity, Gulf Coast Region, Texas and Louisiana. Emerg Infect Dis. 2004;10:1649–52. 15498169 10.3201/eid1009.040203 PMC3320313

[11] Tesh RB, Travassos da Rosa APA, Guzman H, Araujo TP, Xiao SY. Immunization with heterologous flaviviruses protective against fatal West Nile encephalitis. Emerg Infect Dis. 2002;8:245–51. 10.3201/eid0803.010238 11927020 PMC2732478

[12] Davis CT, Beasley DCW, Guzman H, Raj P, D'Anton M, Novak RJ, Genetic variation among temporally and geographically distinct West Nile virus isolates collected in the United States, 2001 and 2002. Emerg Infect Dis. 2003;9:1423–9. 14718086 10.3201/eid0911.030301 PMC2585144

[13] Huelsenbeck JP, Ronquist FR. MRBAYES: Bayesian inference of phylogenetic trees. Bioinformatics. 2001;17:754–5. [cited 2005 June 14]. Program available at http://mrbayes.csit.fsu.edu/download.php 10.1093/bioinformatics/17.8.754 11524383

[14] Swofford DL. PAUP: Phylogenetic analysis using parsimony (and other methods). Version 4. Sunderland (MA): Sinauer Associates; 2002.

[15] Beasley DWC, Davis CT, Guzman H, Vanlandingham DL, Travassos da Rosa A, Parsons RE, Limited evolution of West Nile virus during its southwesterly spread in the United States. Virology. 2003;309:190–5. 10.1016/S0042-6822(03)00150-8 12758166

